# The Effect of Longer versus Shorter Duration of Supervised Physiotherapy after ACL Reconstruction on the Vertical Jump Landing Limb Symmetry

**DOI:** 10.1155/2018/7519467

**Published:** 2018-04-23

**Authors:** Aleksandra Królikowska, Andrzej Czamara, Łukasz Szuba, Paweł Reichert

**Affiliations:** ^1^The College of Physiotherapy in Wroclaw, Kościuszki 4, 50-038 Wroclaw, Poland; ^2^The Center of Rehabilitation and Medical Education, Kościuszki 4, 50-038 Wroclaw, Poland; ^3^Division of Sports Medicine, Department of Physiotherapy, Faculty of Health Sciences, Wroclaw Medical University, Bartla 5, 51-618 Wroclaw, Poland

## Abstract

The study investigated the vertical jump landing limb symmetry after ACLR between a group of patients receiving a longer supervised physiotherapeutic procedure and following a shorter supervised physiotherapy. Group I (*n* = 20) and Group II (*n* = 15) were males averagely 30 weeks after ACLR. The time since ACLR in both groups (Group I, 27.95 ± 8.26 weeks; Group II, 32.47 ± 7.74 weeks) was insignificant, although the duration of supervised physiotherapy between the two groups (Group I, 27.9 ± 8.26 weeks; Group II, 11.28 ± 8.20 weeks) significantly differenced. Group III (*n* = 20) were controls. Two-legged and one-legged vertical jumps landing vertical ground reaction force (VGRF) were bilaterally measured in all groups using force plates. The intragroup comparison of two-legged jump landing VGRF revealed *p* = 0.01 between the involved and uninvolved limbs in Group II. The intergroup comparison revealed *p* ≤ 0.001 in the two-legged vertical jump between Groups II and III, and I and II. The one-legged limb symmetry was comparable in studied groups. In the group following shorter supervised physiotherapy, the two-legged landing limb symmetry was on a worse level than in the group of patients receiving fully supervised procedure and healthy individuals. A fully supervised postoperative physiotherapy is more effective for improving two-legged vertical jump landing limb symmetry.

## 1. Introduction

The reported incidence of 0.7 to 2.5 tears per 1000 athletic exposures in the young and athletically active people makes the anterior cruciate ligament (ACL) the most frequently being injured of all knee ligaments [1]. For patients with functional knee instability and wishing to return to cutting and pivoting sports, the arthroscopic reconstruction of the ligament remains a gold standard of treatment [2]. Even though there exist a large number of standardized postoperative protocols, it is still impossible to find consensus regarding an optimal rehabilitation program to safely and effectively return athletes to their preinjury level of sport [3].

Another controversy remains about achieving satisfactory outcome after ACL reconstruction undergoing home-based, unsupervised physiotherapy procedure, without the direct supervision of a physiotherapist [4–7]. However, to our knowledge, none of those studies involved vertical ground reaction force (VGRF) measurements during one-legged and two-legged vertical jump landing, and what is also important, it is hard to find any studies comparing their results to a group of healthy individuals.

The aim of the study was to investigate the vertical jump landing limb symmetry at seven months after ACL reconstruction between a group of patients receiving a longer supervised physiotherapeutic procedure and a group of patients who followed a shorter supervised physiotherapy. A research hypothesis was formulated that there would be no differences in the vertical jump landing limb symmetry between the two groups of patients.

## 2. Materials and Methods

The experiment gained approval number 1/2012 of the State Committee for Scientific Research of the College of Physiotherapy in Wroclaw, Poland, and was conducted according to the ethics guidelines and principles of the Declaration of Helsinki. All the participants that took part in the study were informed about the purpose and method of research and signed their informed consent form to participate in the study.

The prospective cohort study was carried out in an academic physiotherapy center and included patients of the center in years 2012–2016.

The study cohort comprised three groups of male participants. Group I (*n* = 20) and Group II (*n* = 15) consisted of patients averagely seven months after ACL reconstruction (ACLR) performed by the same team of three surgeons. Group III consisted of 20 volunteers without known orthopaedic problems and matched with ACLR patients in terms of gender, age, body mass, and body weight. There were no statistically significant differences between the three groups in mean body mass and body height. Comparison of age revealed a statistically significant difference between Groups II and III, although the mean age was in the range of 20–30 years in all groups (Table 1).

At the time of the performed assessment, the time since ACLR in both groups of post-ACLR participants (Group I, 27.95 ± 8.26 weeks; Group II, 32.47 ± 7.74 weeks) was statistically insignificant (*p* = 0.11); however, there were statistically significant differences in the postoperative supervised physiotherapy duration between Group I (27.9 ± 8.26 weeks) and Group II (11.28 ± 8.20 weeks). The characteristic of studied groups was presented in Table 1.

### 2.1. Inclusion and Exclusion Criteria

The initial sample of patients that had started the postoperative procedure in the academic center in years 2012–2016 was 50. Participants were recruited to particular study groups based on all of the following inclusion criteria and excluded from the study if they met at least one of the exclusion criteria. Inclusion criteria for Groups I and II were male, unilateral intra-articular transtibial ACLR with the use of autologous ipsilateral semitendinosus (ST), or combined ST and gracilis graft; no additional injuries of the involved and/or uninvolved lower limb or postoperative complications between the time of surgery and performed measurements; and a positive result in the orthopaedic examination described below (grade A, normal). Additional inclusion criteria were for Group I, systematic participation in all four stages of the postoperative physiotherapeutic procedure in the rehabilitation center where the study was carried out, and for Group II, participation in the postoperative physiotherapeutic procedure that ended in the 12th postoperative week at the latest. The inclusion criterion for Group III was male and absence of musculoskeletal injuries in the past. Exclusion criteria for Groups I and II were female (*n* = 5), extra-articular knee surgery (*n* = 0); total resection of medial meniscus (MM) and/or lateral meniscus (ML), *n* = 0; MM/ML transplant (*n* = 0); contralateral graft/patella-tendon graft/quadriceps graft/allograft/other than hamstrings graft used for the ACLR (*n* = 5); posterior cruciate ligament and/or medial- and/or lateral-contralateral ligament repair (*n* = 0); extensor mechanism surgery (*n* = 0); patellofemoral surgery other than cartilage debridement (*n* = 0); articular cartilage injury grade 3 and/or 4 according to the International Cartilage Repair Society (*n* = 5); osteochondritis dissecans lesions (*n* = 0); additional injuries of the involved and/or uninvolved lower limb or postoperative complications occurring between time of surgery and performed measurements (*n* = 0); and grade B, C, or D obtained in the orthopaedic examination described below (*n* = 0).

### 2.2. Surgical Procedures

In Group I, 19 participants underwent single-bundle (SB) and one participant double-bundle (DB) ACLR. Mitek Rigidfix (*n* = 1), Position (*n* = 5), and Endobutton (*n* = 14) were used. MM injury (*n* = 3) and ML injury (*n* = 3) accompanied the ACL injury. The MM was partially resected (*n* = 2) and in one case was sutured. In three cases the ML was partially resected. In Group II, 14 participants underwent SB ACLR and one participant underwent DB ACLR. The All Press-Fit was used in two of the participants and the Position in six. In the remaining cases, the Endobutton was used. In four cases MM injury accompanied the ACL injury. The MM was partially resected in two cases, sutured in one case, and shaved in one case.

### 2.3. Postoperative Physiotherapeutic Procedure in Group I

The participants from Group I underwent four stages of physiotherapeutic procedure led by the authors of the study and based on a physiotherapy protocol for patients after ACLR [8–10]. Stage I lasted from the 1st to the 5th week, stage II from the 6th to the 12th week, stage III from the 13th to the 20th week, and stage IV from the 21st week until 6–8 months postoperatively. A brief characteristic of a fully supervised, standardized physiotherapeutic procedure carried out in Group I was presented in Table 2. The patients took part in the vertical jump landing VGRF measurements in the end of the last stage of physiotherapy.

### 2.4. Postoperative Physiotherapeutic Procedure in Group II

The participants from Group II underwent on average only two first stages of the supervised postoperative procedure being described in Table 2. For the reasons independent from their surgeons and physiotherapist, they refused to continue the supervised physiotherapy. The patients were informed about the main goals and characteristic of the last stages of physiotherapeutic procedures, and they continued the physiotherapy as a home-based one, without being supervised by a physiotherapist. They were asked to take a part in the vertical jump landing VGRF measurements after six months from reconstruction.

### 2.5. Orthopaedic Examination

The participants after ACLR were included or excluded from the study based on the orthopaedic examination and history. According to the 2000 IKDC Knee Examination Form the generalized laxity, alignment, patella position, patella subluxation/dislocation, and range of motion were assessed. Evaluation of effusion, passive motion deficit, manual ligament, and harvest site pathology was also performed. Compartment findings were assessed. The results were assessed using a four-grade scale [11].

### 2.6. Measurement of VGRF during Two-Legged and One-Legged Vertical Jumps Landing

In the three studied groups, the measurement of VGRF during two-legged and one-legged vertical jumps landing was performed by using the MTD-Balance system (MTD Systems, Neunburg v. Wald, Germany) containing two force plates for right and left legs separately. The peak VGRF during landing was analyzed. The methodology of VGRF measurements using the MTD-Balance system was based on the study by Czamara [12].

Prior to measurements, the subjects warmed up on a cycloergometer. The examiner instructed the participant on the proper technique of two-legged and one-legged jumping. The participant performed a few trials until he felt comfortable with the protocol. The command “start” given by the examiner initiated the participant's continuous jumping, and the command “stop” ended it. Each jump was performed in the upright position. The protocol did not allow for countermovement, and arm movement during the jumps was restricted.

First, VGRF values during two-legged vertical jumps landing were measured. At the beginning of the measurement the participant placed his right foot on the middle of the right force plate and left foot on the middle of the left plate. The participant then performed six to 10 continuous two-legged vertical jumps. Second, VGRF values during one-legged vertical jumps landing were measured, starting with the uninvolved leg in Groups I and II and with the right leg in Group III. The participant placed the foot of the studied leg in the middle of the force plate and performed six to 10 continuous one-legged jumps. The second leg was flexed 90° at the knee joint. Measurement was then performed for the second leg in the same way.

### 2.7. Intrarater and Interrater Test–Retest Reliability of VGRF Measurement during Two-Legged and One-Legged Vertical Jumps Landing

Prior to the study the intrarater and interrater test–retest reliability of VGRF measurements during two-legged and one-legged vertical jumps landing using the MTD-Balance system was evaluated. The peak landing VGRF was recorded for analysis. Five males without prior known orthopaedic problems were recruited from the student population of the college where the study was conducted (age 23.00 ± 2.74 years, body mass 82.80 ± 9.73 kg, and body height 184.60 ± 6.88 cm) and participated in the measurement of VGRF during one-legged and two-legged vertical jumping landing according to methodology presented above. The interval between measurements was 7 days. The first two measurements were performed by the same examiner and the third by a different examiner. The results were statistically analyzed by means of the intraclass correlation coefficient (ICC). The test–retest revealed “excellent” intrarater reliability for two-legged jumping landing (ICC = 0.84 for the right limb and 0.96 for the left) and one-legged jumping landing (ICC = 0.96 for the right limb and 0.95 for the left). The interrater reliability was “good” for two-legged jumping landing (ICC = 0.67 for the right limb and 0.69 for the left) and “excellent” for one-legged jumping landing (ICC = 0.94 for the right limb and 0.95 for the left) [13]. In accordance with the test–retest results, all the measurements in the study were performed by the same examiner. To avoid any bias, the results of the measurements were analyzed by an independent researcher.

### 2.8. Statistical Analysis

Statistical analysis was performed with IBM SPSS Statistics 20. For each participant, the mean of obtained peak landing VGRF of executed bilaterally measured one-legged vertical jumps landing and the mean of two-legged jumps landing were calculated separately for each leg and then normalized to body mass. The limb symmetry index (LSI) for each participant in Group I and Group II was calculated as the mean score of the peak landing VGRF obtained by the involved leg divided by the mean score of the VGRF of the uninvolved leg, with the result multiplied by 100. In Group III, the LSI for each participant was calculated as the mean score of the peak landing VGRF obtained by the right leg divided by the mean score of the VGRF of the left leg, with the result multiplied by 100. The LSI was used to define limb symmetry deficits with higher LSI values indicating smaller deficits. The mean value (*x*) and standard deviation (SD) of relative VGRF and LSI were calculated for each study group. Data distributions were tested for normality by the Shapiro–Wilk test. For the intragroup comparison of normalized to body mass VGRF values, parametric Student's *t*-test for dependent samples was used. One-way analysis of variance, the one-way ANOVA, was used to assess the significance of differences between LSIs of the three studied groups. In cases where the one-way ANOVA revealed *p* < 0.05, Tukey's post hoc test was used. Differences were considered significant at the level of *p* < 0.05. ICCs according to Shrout and Fleiss model 2 were calculated to compare the data obtained in test–retest reliability conducted prior to the study [14]. The ICC was interpreted in accordance with Cicchetti and Sparrow, as follows: ICC < 0.40 poor reliability, ICC 0.40–0.59 fair reliability, ICC 0.60–0.74 good reliability, and ICC ≥ 75 excellent reliability [13].

## 3. Results

The intragroup comparison of two-legged vertical jump landing peak VGRF values normalized to body mass revealed statistically significant differences between the involved and uninvolved leg in Group II (Table 3). The values in Groups I and III did not differ significantly between the two studied limbs (Table 3). Relative peak VGRF obtained in the one-legged vertical jump landing did not differ significantly between the involved and uninvolved leg in Groups I and II and between the right and left leg in Group III (Table 3).

The intergroup comparison of LSI with the use of one-way ANOVA presented in Table 4 revealed statistically significant differences in two-legged vertical jumps landing between the three measured groups. Post hoc testing revealed a significant difference between Groups II and III, and Groups II and I on comparison of LSI values. The comparison of LSI for one-legged vertical jumps landing showed no statistically significant differences between the three studied groups; thus the post hoc test was not needed. It is worth of notice that while the one-legged LSI in both ACLR groups (Group I, LSI = 98; Group II, LSI = 93) was close to LSI in healthy individuals (LSI = 99) indicating limb symmetry deficits less than 10%, the two-legged LSI indicated limb symmetry deficits less than 10% only in the Groups I and III (LSI = 99, and LSI = 100 consecutively) and 17% deficit in Group III (LSI = 83).

## 4. Discussion

The present study indicated that, in the group with shorter supervised physiotherapeutic course after ACL reconstruction, the two-legged landing limb symmetry at averagely seven months postoperatively was on a worse level than in the group of patients receiving fully supervised physiotherapy procedure and in the healthy individuals. The finding suggests that fully supervised postoperative physiotherapeutic procedure is more effective for improving two-legged vertical jump landing limb symmetry. In terms of one-legged vertical jump landing limb symmetry the patients undergoing shorter supervised physiotherapy achieved comparable outcomes with patients who underwent longer supervised physiotherapy and healthy individuals. All in all, the hypothesis of the study was accepted only partially.

The comparison of outcomes of unsupervised versus supervised postoperative physiotherapeutic procedure following ACL reconstruction remains controversial. There are many studies confirming lack of differences for the main outcome variables between patients being supervised by a physiotherapist and patients undergoing home-based procedure, starting from the assessment performed three months [15] and six months postoperatively [16], at one year [7, 17] up to two-four years postoperatively [18, 19]. The authors considered home-based physiotherapy as a feasible, safe, effective method of treatment providing significant cost savings. The studies mostly concerned clinical and functional outcome based on anterior tibial translation, ROM, thigh circumference, muscle strength, hopping tests, and knee scores [15–19]. Some authors have also studied the usefulness and safety of the Nintendo Wii in rehabilitation after anterior cruciate ligament reconstruction [20]. The good results are being explained by some authors by patients' motivation and taking responsibility for their own progress [7]. However, none of the studies concerning the home-based physiotherapy issue involved VGRF measurements during one-legged and two-legged vertical jump landing with the use of force plates, and what is also important, there are not many studies comparing the results to a group of healthy individuals.

An interesting finding of the study is that in contrast to the two-legged jump landing limb symmetry, the one-legged jump landing limb symmetry was similar in the three studied groups, implying that regaining the two-legged jump landing limb symmetry is more difficult than regaining the one-legged jump landing symmetry. In light of the risk of contralateral ACL injury when returning after ACLR to sport involving jumping, pivoting, and side-stepping of the knee [21] and suggestions that the injuries might be a result of “favoring” the reconstructed knee while placing the contralateral limb under greater stress [21], decision-making regarding a return to sport would be more precise if focused on objective measures of functional status to guide such a decision. In addition, analysis of two-legged vertical jump landing LSI rather than one-legged LSI before an ACLR patient's return to high-level sport activities may have significant influence on reducing the risk of subsequent injuries, especially those involving the contralateral limb. Protection of the involved limb by shifting the body weight to the contralateral was also confirmed in an analysis of drop jumping in ACLR patients [22]. The study conducted in 2011 by Czamara involved the assessment of landing symmetry in ACLR patients who underwent the same physiotherapeutic procedure, but the results were not compared with those of healthy individuals [12]. However, the author also noted that the limb symmetry in two-legged vertical jumps occurred later than the symmetry in one-legged vertical jumps [12].

Patients demonstrating limb symmetry before they return to high-level sport activities may have a significantly reduced risk of subsequent ACL injuries [23, 24]. Balance training and two-legged landing technique drills teach patients a proper movement pattern, especially when considering a return to game-based sports. Unexpected situations such as tackles, foul play, or specific sport tasks require sound landing techniques when distracted at the moment of flight. Thus the main goal of the two last stages of the physiotherapeutic procedure for Group I [8, 9] was to gradually and safely develop basic skills to prepare the post-ACLR patient for physical activity at the level of recreational or competitive sport. The third stage started in the 13th week and lasted until the 20th week postoperatively. The procedure involved exercises of the involved knee extensors, first under isometric conditions with resistance and then, from the 16th postoperative week, under isokinetic conditions, in addition to strength training of other muscle groups of the lower limb, pelvis, and trunk. During this stage of rehabilitation, running on a treadmill was also included. Patients performed discipline-specific exercises, plyometric exercises, and functional training. The fourth stage, starting in the 21st week and lasting until 6–8 months postoperatively, was focused on restoration of speed, power, and agility, and glycolytic linear conditioning [8, 9]. By this time post-ACLR patients undergoing longer-term physiotherapy had performed at least 6 weeks of developmental stretch shortening cycle plyometric exercises, which may have caused high limb symmetry values for two-legged jumps landing. In regaining limb symmetry in jumping, the crucial role of the stages of physiotherapy lasting from the 12th week to 24–36 weeks postoperatively was also studied by other authors [12, 22].

The clinical relevance of the study is based on showing the benefit in fully supervised physiotherapy after the ACL reconstruction in balancing the limb symmetry in the two-legged jump landing in the level of healthy individuals. When planning a physiotherapy procedure following the reconstruction the clinician must remember several critical components conditioning the regaining of the limb symmetry during two-legged tasks.

One of the main limitations of the study is a relatively small sample. In the future longer follow-up is needed as only seven-month observation may be also considered as a limitation of the study. The findings of the study should be confronted with a more comprehensive assessment involving strength measurement, functional assessment, and proprioception deficits analysis. It would be also crucial to find out if the observed altered limb symmetry during the two-legged jump landing may predispose patients after ACLR to potential reinjuries of ipsilateral and contralateral limbs.

## 5. Conclusion

In the group that underwent shorter supervised physiotherapeutic course following ACL reconstruction, the two-legged landing limb symmetry at seven months postoperatively was on a worse level than in the group of patients receiving fully supervised physiotherapy procedure and in the healthy individuals. A fully supervised postoperative physiotherapeutic procedure is more effective for improving two-legged vertical jump landing limb symmetry. In terms of one-legged vertical jump landing limb symmetry, the patients undergoing shorter supervised physiotherapy achieved comparable outcomes with patients who underwent longer supervised physiotherapy and healthy individuals.

## Figures and Tables

**Table 1:** Characteristics of study groups.

	Group I (*n* = 20)		Group II (*n* = 15)		Group III (*n* = 20)		*p*
	*x*	SD	*x*	SD	*x*	SD	
Age (years)	25.85	5.20	27.33	6.26	22.55	1.82	0.02^*∗*^
Body mass (kg)	85.60	11.33	79.60	9.52	77.20	9.92	0.07
Body height (cm)	184.80	8.61	179.33	5.90	181.65	7.22	0.11
Time since ACLR (weeks)	27.95	8.26	32.47	7.74	–	–	0.11
Duration of postoperative physiotherapy (weeks)	27.95	8.26	11.29	8.20	–	–	≤**0.001**
Involved leg: right/left (*n*)	11/9		7/8		–	–	–

*n*, number of individuals; *p*, significance level; SD, standard deviation; *x*, arithmetic mean. ^*∗*^The post hoc test revealed difference between Groups II and III. Statistically significant *p* values are in boldface.

**Table 2:** Characteristics of particular stages of postoperative procedure carried out in the ACLR groups.

Postoperative physiotherapy stages	Characteristics of particular stages
Stage I From the 1st to the 5th week Main goals: reducing pain and effusion, knee ROM, and gait restoration	Ice packs replaced after couple days with local cryotherapy; continuous passive motion (CPM) knee exercises, mobilization of the patellofemoral joint and soft tissue techniques, electrostimulation of vastus medialis, and magnetic field were applied. Consecutively, closed kinetic chain (CKC) proprioceptive exercises, isometric tensioning of the knee extensors and flexors muscles, followed by isometric exercises with manually dosed resistance. The exercises of muscle groups distant to the affected area, including the uninvolved lower extremity, the upper extremities, and the trunk.

Stage II From the 6th to the 12th week Main goals: improving gait pattern, proprioceptive stimulation	Mobilization of the patellofemoral joint, electrostimulation of vastus medialis, CKC proprioceptive exercises, isometric exercises with manually dosed resistance of the knee flexors and extensors muscles; the exercises of the uninvolved lower extremity, the upper extremities, and the trunk were continued. Treadmill walking; cycloergometer; proprioception exercises progressed to being performed on a soft surface; step-up exercises, two-legged and consecutively one-legged partial squats on a collapsible surface, and concentric and eccentric exercises for the ischiotibial muscles were added.

Stage III From the 13th to the 20th week Main goals: reducing strength asymmetries, teaching proper landing technique, and running for general endurance training	Isometric exercises with partial resistance from the extensor muscles of the involved knee; low intensity plyometric exercises and landing technique reeducation; functional training with movement pattern corrections; complex core exercises were added. In the end of the stage, strength training under isokinetic conditions and treadmill running for general conditioning were introduced. The training was stopped when the patient experienced subjective or objective symptoms of fatigue or pain.

Stage IV From the 21st week until 6–8 months Main goals: practicing complex movement patterns, strength, power, and specific endurance training	Concentric-eccentric lower limb exercises. Discipline-specific exercises without contact, unilateral plyometrics practice, agility drills, and complex neuromuscular training were added. Specific endurance exercises were introduced. The training was stopped when the patient experienced subjective or objective symptoms of fatigue or pain.

**Table 3:** Intragroup comparison of normalized to body mass peak vertical ground reaction force values obtained in two-legged and one-legged vertical jumps.

	Studied limb	Two-legged vertical jump		One-legged vertical jump	
		*x*	SD	*x*	SD
Group I	Involved	20.71	4.39	26.51	4.17
	Uninvolved	21.11	4.38	27.06	4.46
	*p*	0.51		0.22	

Group II	Involved	18.57	3.50	24.92	4.93
	Uninvolved	22.82	4.40	26.91	4.36
	*p*	**0.01**		0.18	

Group III	Right	20.06	4.31	27.53	4.81
	Left	20.56	5.62	27.95	4.74
	*p*	0.46		0.27	

*n*, number of individuals; *p*, significance level; SD, standard deviation; *x*, arithmetic mean. Peak vertical ground reaction force normalized to body mass (N/kg). Statistically significant *p* values are in boldface.

**Table 4:** The intergroup comparison of two-legged and one-legged vertical jump landing limb symmetry index.

	Vertical jump landing limb symmetry index							
	Group I		Group II		Group III		*p* ^1^	*p* ^2^
	*x*	SD	*x*	SD	*x*	SD		
Two-legged jump	99.25	6.95	83.03	16.16	100.89	15.99	≤**0.001**	Group I versus Group III **p** = 0.002
								Group II versus Group III **p** = 0.001
								Group I versus Group II *p* = 0.924

One-legged jump	98.23	6.96	93.52	16.00	99.35	5.84	0.231	-

*p* ^1^, significance level (one-way ANOVA test); *p*^2^, significance level (post-hoc Tukey's test ); SD, standard deviation; *x*, arithmetic mean. Statistically significant *p* values are in boldface.
